# The only emperor is the emperor of ice-cream

**DOI:** 10.3201/eid1706.AC1706

**Published:** 2011-06

**Authors:** Polyxeni Potter

**Affiliations:** Author affiliation: Centers for Disease Control and Prevention, Atlanta, Georgia, USA

**Keywords:** art science connection, emerging infectious diseases, art and medicine, Max Weber, Figures, cubism, modernism, HIV/AIDS, about the cover

**Figure:**
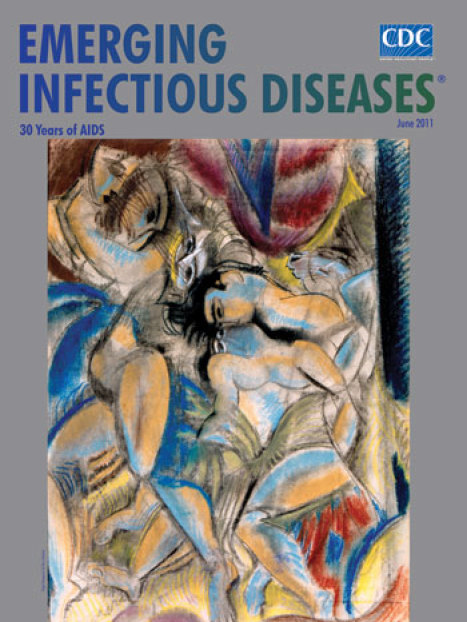
**Max Weber (1881–1961) *Figures* (c. 1914) Pastel on paper (61 cm × 45.7 cm).** High Museum of Art, Atlanta. Gift in memory of Louis Regenstein by his wife Helen and sons Lewis and Kent.

―Wallace Stevens

“So many, I had not thought death had undone so many.” These words about the precariousness of the early years of the 20th century referred to lives lost in war. T.S. Eliot and other poets and writers, along with many in the arts and sciences, were grappling with growing cities and their beleaguered populations and with technological advancements that allowed the killing of unprecedented numbers of soldiers in battle. At the same time, they were swept in a wave of creativity and change centered in Paris and spreading all over the world. These were the times of Albert Einstein, James Joyce, Diego Rivera, Igor Stravinsky, and many others, who were leading scientific, literary, and artistic trends under the broad umbrella of modernism as they tried to “make it new” with outlandish forms and styles.

In the United States, the effects of modernism also permeated technology, photography, film, and dance. American painters, many of whom had gone to Europe, were familiar with modern styles, which they absorbed and carried to their own continent. Max Weber, along with Marsden Hartley, John Marin, Georgia O’Keefe, and others, was part of this avant garde.

Born in Bialystok, then Russia, Weber immigrated to the United States with his family, which settled in New York City when he was 10. He attended public schools and studied art at the Pratt Institute in Brooklyn. He apprenticed with painter and printer Arthur Wesley Dow, who ahead of his time advocated examining visual relationships between forms rather than working solely with objects and believed in “filling a space in a beautiful way” rather than recreating nature. Weber went off to teach in Virginia and Minnesota to finance travel to Paris, where for a time he studied at the Académie Julian and received classical training under painter Jean-Paul Laurens. While abroad, the artist also traveled to Italy, Holland, and Spain.

In Paris, he made the right connections. He knew Cézanne; Gauguin; and Picasso, who along with Braque pioneered cubism, a movement that quickly spread into sculpture, architecture, literature, and music, moving beyond single point perspective to capture objects from many angles at once, forever changing the way we view the world. He became close friends with Henri Rousseau and was involved in organizing a course led by Matisse, a transformative experience in his handling of color. He exhibited in major Salons.

In cubism objects are broken into parts, analyzed, and reassembled, their form enriched in the process. Surfaces often intersect at unusual angles and with the background, muddling traditional perception of depth. These radical concepts immediately resonated with poets and writers, many of whom were also in Weber’s Parisian circle: Guillaume Apollinaire, Robert Delaunay, Gertrude Stein. Like the art of modernism, its poetry was abstract and multifaceted, complex and demanding. In the United States some of William Faulkner’s work has been interpreted in cubist terms, as well as that of Wallace Stevens, who wrote “Thirteen Ways of Looking at a Blackbird” and other poems along these lines.

Back in New York, Weber experimented with cubism as he worked toward his own style. He benefited from a brief association with talented photographer and art publisher Alfred Stieglitz and his gallery 291, which promoted photography as a legitimate method of image making and advocated modernism. In Stieglitz’s journal Camera Work, he expanded on his notion of the fourth dimension―“The consciousness of a great and overwhelming sense of space-magnitude in all directions at one time…. It is real and can be perceived and felt.”

Cinematic innovations fueled Weber’s experiments with movement and time, as seen in *New York at Night* (1915), a painting that conveyed the speed, action, and dynamic energy of the city. His intent was to express “not what I see with my eyes but with my consciousness… mental impressions, not mere literal matter-of-fact copying of line and form. I want to put the abstract into concrete terms.” He converted everyday experiences, such as walking into a dark auditorium to attend a lecture, into iconic abstractions: “The late hastening visitor finds himself in an interior of plum-colored darkness… upon which one discerns the focusing spray-like yellowish-white light, the concentric, circular rows of seats, a portion of the screen.” This experience of the dark turned into *Slide Lecture at the Metropolitan Museum* (1916).

After the end of his association with Stieglitz, Weber supported himself by teaching art history, appreciation, and design at the Clarence H. White School of Photography and at the Art Students League. Although in the end of his career he turned to less radical subjects and forms, he continued to advance the cause of modernism until his death in Great Neck, Long Island.

One of the first American painters to understand and embrace cubist analysis and restructuring, Weber was able to integrate it into the contemporary American scene: skyscrapers, airplanes, subways, lights, the movies. Initially rejected for this revolutionary work, he is now recognized for expressing the ideals and concerns of his times.

While the beginning of the 20th century was marred by rapid change and the deaths of war, its end was no less shaken by globalization, social and political strife, and on the public health front—brought on by unprecedented industrial growth, ecologic and demographic changes, and explosive travel—one of history’s worst pandemics, HIV/AIDS. A modern plague, this one had all the social and economic markers of previous scourges and a death toll of millions.

*Figures*, on this month’s cover, captures both the complexity of the scientific challenge of this unknown and lethal disease and the massive human loss. Oddly reminiscent of the pathetic piles of bodies in carts and public graves during the medieval plague pandemic, Weber’s fractured figures, lyrical but lifeless, are frozen in time. Masked and mysterious, they seem neither critical of their demise nor passive and acquiescent. Like Wallace Stevens’ poetic characters, whether blackbirds or humans as in “The Emperor of Ice Cream,” they simply are. A tangled human web, they array nothing. But their closeness poignantly suggests that we are all in this together, sharing the human condition, the inevitability of death—in this case, an early and cruel one.

In regards to HIV/AIDS, modernism seeped into science. With uncharacteristic speed, the dreaded plague largely found its match in a multidisciplinary but uniquely integrated public health approach combining human rights advocacy and prevention measures and epidemiologic and surveillance data with virology and immunology. And although the puzzle awaits final solution, clinical therapies and rigorous health education have extended the lives of HIV-infected persons, with public health reaching the same conclusion as art and poetry: The only element of value is to “be” alive. Prolong and embrace it. All other considerations only “seem” important. Or as Stevens put it, “Let be be the finale of seem / The only emperor is the emperor of ice cream.”
